# The Lyon Clinical Olfactory Test: Validation and Measurement of Hyposmia and Anosmia in Healthy and Diseased Populations

**DOI:** 10.1155/2011/203805

**Published:** 2011-10-20

**Authors:** Catherine Rouby, Thierry Thomas-Danguin, Michel Vigouroux, Gabriela Ciuperca, Tao Jiang, Jérôme Alexanian, Mathieu Barges, Isabelle Gallice, Jean-Louis Degraix, Gilles Sicard

**Affiliations:** ^1^Centre de Recherche en Neuroscience de Lyon, CNRS UMR 5292-INSERM U1028 and Université Claude Bernard Lyon 1, Université de Lyon, 69366 Lyon Cedex 07, France; ^2^Centre des Sciences du Goût et de l'Alimentation, UMR 6265 CNRS, UMR 1324 INRA, Université de Bourgogne, 21065 Dijon Cedex, France; ^3^Institut Camille Jordan, CNRS UMR 5208, Université Claude Bernard Lyon 1, Université de Lyon, 69622 Villeurbanne, France; ^4^Pôle de Gérontopsychiatrie, Hôpital Saint Jean de Dieu, 290 route de Vienne BP 8252, 69355 Lyon Cedex 08, France; ^5^Audiologie et Explorations Oro-Faciales, Hôpital Edouard Herriot, Université Claude Bernard Lyon 1, Université de Lyon, 69437 Lyon Cedex 03, France; ^6^Laboratoire de Neurobiologie des Processus Mnésiques, UMR 6149 CNRS, Université de Provence, 13331 Marseille Cedex 03, France

## Abstract

The LCOT is a self-administered test designed to assess olfactory deficits. Altogether, 525 subjects contributed to the validation. Elderly participants were well represented in this sample. In a validation study (study 1), 407 healthy and 17 anosmic volunteers between 15 and 91 years of age underwent threshold, supraliminal detection, and identification testing. Cutoff values for normosmia and hyposmia were calculated and applied in a second study in a group of patients with smell complaints and in a group of Alzheimer patients with age-matched controls. Incidence of smell deficit was estimated at 5.6% in the healthy population of study 1, and at 16% in the elderly control group of study 2. Assessment of the ability of each subtest to discriminate between groups showed that LCOT is relevant to differentiating between perception and identification deficits and between Alzheimer's and hyposmic patients.

## 1. Introduction

Smell is a key to our relationship to food, approach/avoidance behavior, and alarm response to dangerous chemicals [1]. Quality of life in general is partly dependent on the ability to smell, as shown by the complaints of patients experiencing loss of olfactory sensitivity: mood swings or depression, and worries about personal hygiene, safety, social interaction, and so forth, (see [2] for a review). Although epidemiological surveys were conducted in Sweden [3], the USA [4], Germany [5], and Australia [6], the frequency of olfactory dysfunction remains poorly documented in the French population. The prime aim of this study was therefore to provide a tool to measure this prevalence.

In recent decades, several olfactory tests were designed in various countries (see [7]). Some measured identification only [8–10], some sensitivity only [11], others combined both [7, 12–15], and one added a discrimination measurement [14]. Thus, the different commercially available tests do not specifically measure the same olfactory competencies, but all are designed to detect hyposmia or anosmia. 

With hyposmia being operationally described as an impairment of both sensitivity and quality perception [12], we wanted a clinical test that would measure both. Each addresses different competencies: whereas sensitivity reflects perceptual processes that do not strongly depend on language abilities, identification relies on language and culture. Cultural variation conditions odor identification, which is based on learning of odors that have become familiar and “ecologically valid” [16]; such familiarity varies from country to country [17–19], as does stimulus typicality for a given target odor [20]. These considerations led Doty et al. [21], for example, to modify the American UPSIT test for use in Asia and Europe. In the European Test of Olfactory Capabilities (ETOC), odorants were selected so as to reduce cultural differences in familiarity across countries [7, 22]. The importance of language in odor perception is well known [23], and a requisite of identification tests is to help identification by providing participants with several names in a forced-choice paradigm [16]. Closer examination of these semantic cues shows that the choice of appropriate labels is a decisive factor in successful identification [24]. Thus, a second prerequisite for our clinical test was the ecological validity of the odorants and of their names for a French population.

 One of the prominent causes of impaired smell ability is aging: this decrease in olfactory function during normal aging is called presbyosmia [25, 26]. It was therefore important to collect data from healthy subjects in all age groups from 15 to 90 years in order to establish normative lifespan data for both sexes. Such control data were required in order to be able to compare patient groups to the general population.

Because clinicians need a short self-administered test of olfactory function, the Lyon Clinical Olfactory Test was designed with 3 main purposes:

1. to describe the sensitivity and identification abilities of the French population by combining subtests based on perceptual and cognitive abilities so as to help orient clinicians towards a central or peripheral hypothesis in case of olfactory loss; the test, therefore, crossed 3 types of measurement: threshold, supraliminal detection, and identification. This approach was intended to allow differentiation between problems of sensitivity and of naming and to be useful in patients with cognitive deficits;
2. to categorize the population into 3 classes (normosmic, hyposmic, and anosmic) by establishing cutoff values;
3. to validate the test and the derived norms by test-retest measurement and application to patient populations: one sample of patients with Alzheimer's disease and another of patients from our smell clinic.

## 2. Materials and Methods

### 2.1. Principle

The Lyon Clinical Olfactory Test (LCOT) is composed of blocks of four 15 mL vials [22, 27, 28]. Only 1 of the 4 contains an odorant, dissolved in odorless mineral oil (Sigma-Aldrich) soaked up on oil-absorbent to avoid any leakage during vial opening and to increase the area of exchange with the air. The other vials are blanks containing only the mineral oil solvent soaked up on the oil-absorbent. The whole procedure is based on 4 alternative forced choices (4-AFC).

Testing consisted of 3 tasks: threshold detection, supraliminal detection, and identification.

Threshold Measurements used 2 different odorants: R-(+)-carvone (minty odor, Sigma-Aldrich) and tetrahydrothiophene (THT, gas odor and Euracli). These familiar and thus “ecological” odorants were used instead of the classical l-butanol because the latter also stimulates the trigeminal nerve [29]. Moreover, using the familiar main gas odor was relevant for the clinicians, who could warn people when they did not detect it. The same threshold determination procedure was used for both compounds: 5 concentration levels were presented, from weakest to strongest (dilution factor: 10), with a forced choice paradigm (ascending staircase 4-AFC procedure). For each block, subjects were told to smell the 4 vials consecutively and to indicate on a response sheet which one smelled strongest or else, if they did not smell any difference between the vials, to guess (4-AFC). No feedback on response correctness was given. The blocks were presented in increasing order of concentration: 10^−6^, 10^−5^, 10^−4^, 10^−3^and 10^−2^ (vol./vol.) for R-(+)-carvone and 10^−7^, 10^−6^, 10^−5^, 10^−4^ and 10^−3^ (vol./vol.) for THT.

Supraliminal Detection and Identification Measurements used a series of 16 odorants, diluted at an easily detectable concentration level (around 10^−2^) (Table 1).

Following the same 4AFC procedure, subjects were to smell a block of 4 vials, detect which vial contained an odor, and then identify the odor by selecting a label among 4 proposed alternatives. For instance, the 4 alternative labels associated with the lavender stimulus were “leather”, “paint,” “lavender,” and “almond”. The test was self-administered. Subjects were given the 26 test blocks (5 for carvone threshold, 5 for THT threshold, and 16 for suprathreshold detection and identification), an instruction sheet, and a response sheet; they recorded their answers by circling their choice (vial code or label) on the response sheet, with their age, gender, smoking habits, and possible nasal diseases. They worked individually under the supervision of a trained experimenter. The time required to open and close the 4 vials and to mark the response on the sheet ensured that there was an interval of at least 45 sec between stimulations, reducing the risk of adaptation. Testing lasted about 30–35 minutes per subject.

### 2.2. Scoring

The threshold score was defined as the lowest odor concentration detected and followed by correct detections. Scores ranged from 5 (when the odor was detected correctly from the weakest concentration) to 1 (when the odor was detected correctly only at the strongest); subjects who failed to detect the strongest concentration were scored 0. Two threshold scores were recorded for each subject: R-(+)-carvone threshold (CT) and THT (gas odor) threshold (GT), both from 0 to 5.

For each subject, supraliminal detection performance (DP) was the number of correct detections (from 0 to 16), identification performance (IP), and the number of correct responses (from 0 to16). The rationale of this supraliminal detection level is that odor identification was scored as correct only when the corresponding odor vial was detected correctly: as the risk of correct detection by chance and of correct identification by chance was for both 1/4, the scoring procedure reduced the probability of identifying an odor by chance to 1/16. 

Moreover, because DP measured a perceptual ability and IP measured a more verbal one, it was hypothesized that the difference between these scores (DP−IP) could reveal some cognitive components of olfactory deficit; a differential score (DD = DP − IP) was therefore calculated (from 0 to 16).

### 2.3. Odorants

Pure chemical compounds were purchased from Sigma-Aldrich. Domestic fuel-oil was purchased from Total. The aromas were kindly provided by Euracli (Chasse-sur Rhône, France). These smelling compounds were selected for their high level of familiarity for a French population [24] (Table 1). They were diluted in mineral oil (Sigma-Aldrich, 1%), except for *ω*-pentadecalactone which was diluted in diethyl phthalate (Sigma-Aldrich, 10%).

## 3. Study 1

The purpose of study 1 was to determine normative scores for normosmic, hyposmic, and anosmic subjects, and to assess test-retest reliability.

### 3.1. Participants

#### 3.1.1. Healthy Volunteers

Participants were recruited from volunteers in public sessions organized by Lyon-1 University. Testing was run in accordance with the Declaration of Helsinki. Volunteers who presented signs of nasal irritation or declared olfactory disorder were excluded. Thus, 407 participants between 15 and 91 years of age were included (Table 2); 92 (23%) were smokers, 221 (54%) had never smoked, and 94 (23%) were exsmokers. The sex ratio was 61% in favor of women.

#### 3.1.2. Anosmic Volunteers

Seventeen anosmic participants (10 women), all volunteers, participated. They were diagnosed as anosmic according to their medical history, following rhinitis, head trauma, or Kallmann syndrome, or without known etiology. Patients with nasal obstruction at time of testing were excluded. Ages ranged between 16 and 70 years.

### 3.2. Test-Retest Sample

Twenty participants (12 women) from 18 to 59 years of age were retested within a 2-month interval. Seventeen belonged to the healthy and 3 to the anosmic sample.

### 3.3. Statistical Analyses

Regressions and ANOVAs were performed using SAS release 9.1.3 (SAS Institute Inc., Cary, NC) and the REG and GLM procedures. The hypothesis tests on the mean of the normal distribution were carried out with the *t*-test function of *R* release 2.12.0 (The *R* Foundation for Statistical Computing).

### 3.4. Results

#### 3.4.1. Healthy Participants

Standardization was based on the results of the 407 healthy participants; Table 3 presents their mean scores for each subtest.

To test whether sex and smoking habits influenced scores on the 5 subtests, 2-way analysis of variance was performed (*Y*_*ij*_ = *μ* + sex_*i*_ + tobacco_*j*_+ (sex *¤* tobacco)_*ij*_ + *ε*_*ij*_, *ε*_*ij*_ *εN*(0, *σ*_2_)).

For all 5 subtests (CT, GT, DP, IP, and DD), the models showed no significance at the 5% level, indicating no influence of sex or smoking habits on test performance.

To assess the influence of age on the scores of healthy participants, regression analysis was performed (*Y*_*i*_ = *a* + *b* · age_*i*_ + *ε*_*i*_, *ε*_*i*_ ~ *N*(0, *σ*_2_)). One regression was carried out for each subtest (CT, GT, DP, IP, and DD). The effect of age was tested using the Fisher statistic ((H_0_): *b* = 0 against (H_1_): *b* ≠ 0). Results are presented in Table 4.

Age had no significant effect on CT and GT threshold scores, but significantly influenced IP, DP, and DD scores (*α* < 0.0001). CT score distribution was not normal: 34 subjects scored 0 or 1; this represented 8.4% of the tested population, in agreement with the percentage of specific hyposmia to R-(+)-carvone in the general population [30]; the CT score was therefore discarded for normative data calculation. As age did not influence GT score, we looked for the lower limit of this subtest score for healthy participants. Given the mean GT score of 3.97, a Student's *t*-test was used to determine whether healthy participants could score lower than 3 (H_0_: GT ≤ 3 against H_1_: GT > 3); results indicated that healthy participants statistically scored at least 4 (df = 406, *t* = 5.9, *P* ≤ 0.0001). As age influenced DP and IP scores, we looked for the lower limit of each subtest score according to age. For DP, a Student's *t*-test was used to determine whether healthy participants between 15 and 71 years of age could score lower than 14 (age ∈ [15 : 71], H_0_: DP ≥ 14 against H_1_: DP < 14); results showed that they detected at least 14 odors (df = 338, *t* = 44.3, and *P* = 1), whereas healthy participants older than 72 years detected at least 13 odors (age ∈ [72 : 91], H_0_: DP ≥ 13 against H_1_: DP < 13; df = 62, *t* = 11.7, *P* = 1).

The same procedure applied to IP scores showed that healthy participants between 15 and 64 years of age identified at least 13 odors (age ∈ [15 : 64], H_0_: IP ≥ 13 against H_1_: IP < 13; *t*-value = 65.9, df = 304, *P* = 1), compared to at least 12 odors between 65 and 74 years of age (age ∈[65 : 74], H_0_: IP ≥ 12 against H_1_: IP < 12; *t*-value = 36.6, df = 52, *P* = 1) and at least 9 odors between 75 and 91 years of age (age ∈ [75 : 91], H_0_: IP ≥ 9 against H_1_: IP < 9; *t*-value = 29.97, df = 48, *P* = 1).

For DD scores, the same procedure indicated that healthy participants between 15 and 61 years of age obtained a DD score ≤2 (age ∈ [15 : 61], H_0_:  DD ≤ 2 against H_1_: DD > 2; *t*-value = −14.4, df = 292, *P* = 1), those between 62 and 71 years ≤4 (age ∈ [62 : 71], H_0_: DD ≤ 4 against H_1_: DD > 4; *t*-value = −6.9, df = 45, *P* = 1) and those between 72 and 91 years ≤7 (age ∈ [72 : 91], H_0_: DD ≤ 7 against H_1_: DD > 7; *t*-value = −13.97, df = 67, *P* = 1).

#### 3.4.2. Anosmic Participants

Mean scores in the anosmic patients group were: GT = 0.88 ± 1.05, DP = 4.71 ± 2.28, IP = 1.42 ± 1.80, and DD = 3.5 ± 1.63. These corresponded to scores expected for random choices. The mean DD score was higher than in the healthy participants sample: that is, most of the items detected were not identified. Due to the small number of patients in anosmic group, the influence of smoking habits and sex was not tested. To assess the influence of age, simple regression analysis was performed (*Y*_*i*_ = *a* + *b* · age_*i*_ + *ε*_*i*_, *ε*_*i*_ ~ *N*(0, *σ*_2_)) for each subtest (GT, DP, IP, and DD) and tested on the Fisher statistic ((H_0_): *b* = 0 against (H_1_): *b* ≠ 0): age was found not to influence scores, as none of the 4 regressions were significant (*α* = 0.05).

The lower limit of each subtest score was determined, regardless of age: anosmics never scored better than 3 for GT (H_0_: GT ≤ 3 against H_1_: GT > 3; df = 16, *t*-value = 8.28, *P* = 1) and did not detect more than 7 odors (H_0_: DP ≤ 7 against H_1_: DP > 7; df = 16, *t*-value = 4.13, *P* > 0.99); on IP, they did not identify more than 5 odors (H_0_: IP ≤ 5 against H_1_: IP > 5; df = 16, *t*-value = 8.2, *P* = 1). These scores were higher than chance, as will be discussed later. DD (i.e., the difference between DP and IP) did not exceed 5 (H_0_: DD ≤ 5 against H_1_: DD > 5; df = 16, *t*-value = 3.92, *P* > 0.99).

As shown in Table 5, for this sample of subjects, the condition, DP ≤ 7, was sufficient to detect anosmics. To detect normosmics, the conditions DP ≥ 14 for ages [15 : 71] and DP ≥ 13 for ages >71 were sufficient. By definition, hyposmics were in between. 

GT score was not included in the definition of normosmia because, according to the confidence limits of the mean, the scores of the healthy sample ranged from 3.10 to 4.84; thus requiring a GT score of 4 for normosmia would have categorized as hyposmic many participants who were within the normal range for the other criteria. 

As an internal validation of the normative data is obtained, the criteria for DP, IP, and DD were applied to both populations (407 healthy participants and 17 anosmic patients), with the following results (Table 6).

Twenty three healthy participants were found to differ from the healthy and anosmic groups and were classified as hyposmic (5.6%). In the anosmic sample, 1 of the 17 patients was classified as hyposmic (5.8%). 

**Figure 1:**
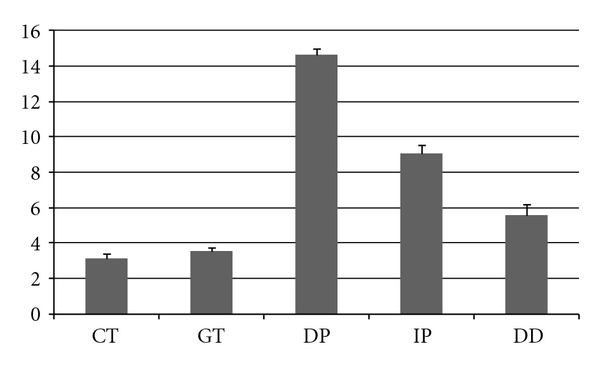
Means and standard errors of scores for the group of healthy participants classified as hyposmic.

Figure 1 presents the mean scores of this hyposmic group, which was composed of 14 women (61%) and 9 men (39%), with a mean age of 62 ± 19 years. 

The smell problems of these 23 subjects did not concern supraliminal detection: they detected only 1 odor less than the whole healthy sample. Their mean thresholds were moderately lower than those of the 407 healthy subjects: CT = 3.09 versus 3.36; GT = 3.52 versus 3.97. Therefore, these 23 subjects seemed to experience mild hyposmia with moderate reduction in sensitivity. Their main smell problem resided in identification performance, which was 5 points less than the sample as a whole (9 versus 14.1), entailing a higher DD score (5.56 versus 1.47).

### 3.5. Test-Retest Reliability

Score repeatability between test and retest was assessed on binomial test. First, the difference between the test and retest scores was calculated for each participant: 0 if the subject obtained the same score twice (good repeatability) or different from 0 if the scores differed. If participants responded randomly on test and retest, the number of 0 differences should follow a binomial distribution with *n* = 20  *P* = 0.2 (in the case of threshold tests, where scores ranged from 0 to 5) and *P* = 0.0625 (in the case of DP and IP, where scores ranged from 0 to 16). With a *P* = 0.05 risk level, where *P* = 0.2, the limit to reject the null hypothesis is ≥9 and, where *P* = 0.06, ≥5. The null hypothesis could thus in the present case be rejected: that is, test and retest showed significant repeatability (subjects' scores were not random) at risk level <0.05 for GT and much less than 0.001 for DP and IP.

### 3.6. Discussion of Study 1

#### 3.6.1. Normative Data

Other studies define hyposmia in terms of the 10th percentile's global score on the olfactory test (combining threshold, discrimination, and identification scores) [5, 31] of the performance of patients identified a priori as anosmic [10] or by comparison (2 standard deviations) with the performance of healthy subjects under 40 years of age [6]. The present study used scores of participants who considered themselves either anosmic or healthy. A statistical approach was used to obtain cut-off values so that participants whose scores fell in between could be considered hyposmic. Thus, 5.6% of the healthy participants without any declared smell problem could be considered hyposmic.

#### 3.6.2. Threshold Tests

There was no significant effect of age on CT or GT scores. Those subtests did not strongly discriminate sensitivity between subjects. This may have been due to the small number of concentrations used and the absence of a staircase procedure such as recommended for precise measurement of thresholds [32]. Another issue was the high threshold found for l-Carvone in 34 subjects, which may be explained by a specific hyposmia, resulting in a bimodal distribution of thresholds [30]. It would then follow that the carvone threshold is not suitable for screening sensitivity in the population as a whole. On the other hand, the threshold for THT (gas odor) alone seemed to be sufficient to identify anosmics, who scored less than 3. In detecting hyposmia, a difficulty arose regarding the cut-off value for GT: a score of 3 would correspond to hyposmia and a score under 3 to anosmia; but requiring a score of 4 or 5 as a condition for normosmia would result in a large proportion of hyposmics because the scores of the healthy sample actually ranged from 3 to 5. The GT score was therefore discarded from the definition of normosmia and used only in defining anosmia.

#### 3.6.3. DP Score

The results showed that supraliminal detection (DP) alone could distinguish normosmics from hyposmics and anosmics: even the oldest normosmics, over 76 years of age, scored higher than 13; anosmic subjects did not obtain scores higher than 7. The one subject who detected 10 odors was therefore classified as hyposmic according to this cut-off score.

The mean performance of anosmics did not differ from chance; some odorants, however (eucalyptol, carvone, and possibly others) included a trigeminal component that could help some anosmics to detect them above chance level (i.e., 4/16).

On the basis of our sample of subjects, detection performance could be considered as sufficient to categorize subjects as normosmic or hyposmic. This is an important finding because this measure is nonverbal, with the advantages of quick diagnosis and of being independent of culture and linguistic knowledge. This allows smell deficits to be diagnosed on a perceptual basis, whatever the language the subject speaks.

#### 3.6.4. IP Score

The identification task (IP score) was more sensitive to age than detection (DP score), indicating a stronger decrease in cognitive than in perceptual aspects of smell. It is acknowledged that odor identification is cognitively demanding [33] and that age impairs both memory and lexical access to odor names [34, 35].

Overall, the results suggest that some subtests were more relevant to detecting smell impairment; this is important for clinicians, who may thus use rapid screening with DP only when they are short of time. The GT score is relevant to confirming anosmia and the IP score to assessing distortions of odor quality.

#### 3.6.5. DD Score

This score also increased with normal aging: from 2 to 7 between 61 and 91 years. This measure of cognitive smell impairment was useful in study 2, in comparison with pathological aging.

#### 3.6.6. Test-Retest

The repeatability of the LCOT was good: at a 0.05 risk level for the GT score and considerably less than 0.001 for the CT, DP, and IP scores.

#### 3.6.7. Internal Validity

Classification of anosmic participants was good, with 5% misclassification, which was the accepted error risk. The classification of healthy participants suggested a hyposmia prevalence of 5.6% in a sample that excluded subjects with smell problems. This is in the same range as in studies using the tenth percentile of the population to define hyposmia; nevertheless, the question remains as to whether this resulted from misclassification by the statistical model (with an error risk of 5%) or from correct classification of participants with true smell deficits. These “healthy” hyposmic participants may have been unaware of their deficit, or may have suspected it and volunteered precisely in order to test their sense of smell. Alternatively, it could be argued that around 10% of the general population is hyposmic and that a figure of 5.6% represents a recruitment bias in study 1, which was based on volunteers.

## 4. Study 2

The validation study provided norms for the healthy sample and for anosmics, categorizing individuals scoring between the cut-off values as hyposmic. Study 2 sought to test the model and cut-off values on 2 pathological samples: patients with Alzheimer's disease (ALZ group) and volunteer smell-clinic patients complaining of smell troubles (PAT group). These patient groups were compared with a group of healthy controls matched for age with the ALZ patients (CONT group). 

### 4.1. Participants

#### 4.1.1. Olfactory-Impaired Patients

The 36 smell-clinic patients (22 women) consulted at the olfaction outpatients department of Edouard Herriot Hospital (Lyon University), France. Mean age was 51 ± 15 years. Subjects with patent nasal obstruction or showing abnormal secretion were excluded.

#### 4.1.2. Alzheimer's Disease Patients and Control Volunteers

The Alzheimer's study was carried out on patients during day hospital care (Saint Jean de Dieu Hospital, Lyon) (ALZ group) and on age-matched control volunteers (CONT group) without known olfactory disorder (often ALZ-group members' spouses or main caretakers). Thirty-three Alzheimer's patients (28 women; mean age, 77 ± 8 years) and 32 age-matched control volunteers (26 women; mean age, 76 ± 7 years) participated. Testing was run by gerontopsychiatrists. Cognitive impairment was measured in patients and control participants using the Mini-Mental State Examination (MMSE, [36]). A CT scan was performed in the Alzheimer's patients to control vascular dementia, which was an exclusion criterion. When demented patients had praxis difficulties, the physician presented the open vials to the patient and asked: “Does this bottle smell or not?” In the identification test, a second set of questions was: “What does it smell of? Does it smell of leather? Does it smell of paint? Does it smell of lavender? Does it smell of almond?”, to which patients could answer yes/no; when patients refused to make any choice between the 4 items, the investigator randomly attributed one of the four answers. As regards cognitive impairment, the MMSE scores of the control (CONT) group were normal (mean ± SD = 29.2 ± 1.2); the ALZ group obtained lower scores with a larger standard deviation (mean ± SD = 13.0 ± 4.0; range, 5/30 to 21/30). Two-way analysis of variance (age, group) indicated a significant difference between ALZ and CONT (*F* = 11.2; *P* < 0.001) and no significant effect of age on MMSE score (*F* = 0.027, *P* = 0.97).

### 4.2. Methods

Statistical analyses were conducted using SAS release 9.1.3 (SAS Institute Inc., Cary, NC). Analysis of variance was performed with the GLM procedure followed by multiple comparison between means (Tukey's HSD correction for multiple comparisons). Age was introduced as a covariate in these analyses. Reported means are least square means.

### 4.3. Results

#### 4.3.1. Subject Classification

According to the cut-off scores, 14 PAT group participants were classified as anosmic (39%), 18 as hyposmic (50%), and 4 as normosmic (11%). According to the same rules, the ALZ group comprised 1 anosmic (3%), 21 hyposmics (64%), and 11 normosmics (33%), and the CONT group 5 hyposmics (16%) and 27 normosmics (84%).

Analysis of variance including age as covariate was carried out for each type of score to assess differences between the 3 groups (CONT, PAT, and ALZ). Results consistently showed a significant group effect (GT: *F*(2,97) = 6.4, *P* = 0.003; DP: *F*(2,97) = 7.2, *P* = 0.001; IP: *F*(2,97) = 27.3, *P* < 0.0001; and DD: *F*(2,97) = 31.1, *P* < 0.0001). Age had only a barely significant effect on GT score (*F*(1,97) = 4.1, *P* = 0.046).

#### 4.3.2. Contribution of Each Score to Group Discrimination

Figure 1 presents the mean scores for each group.

**Figure 2:**
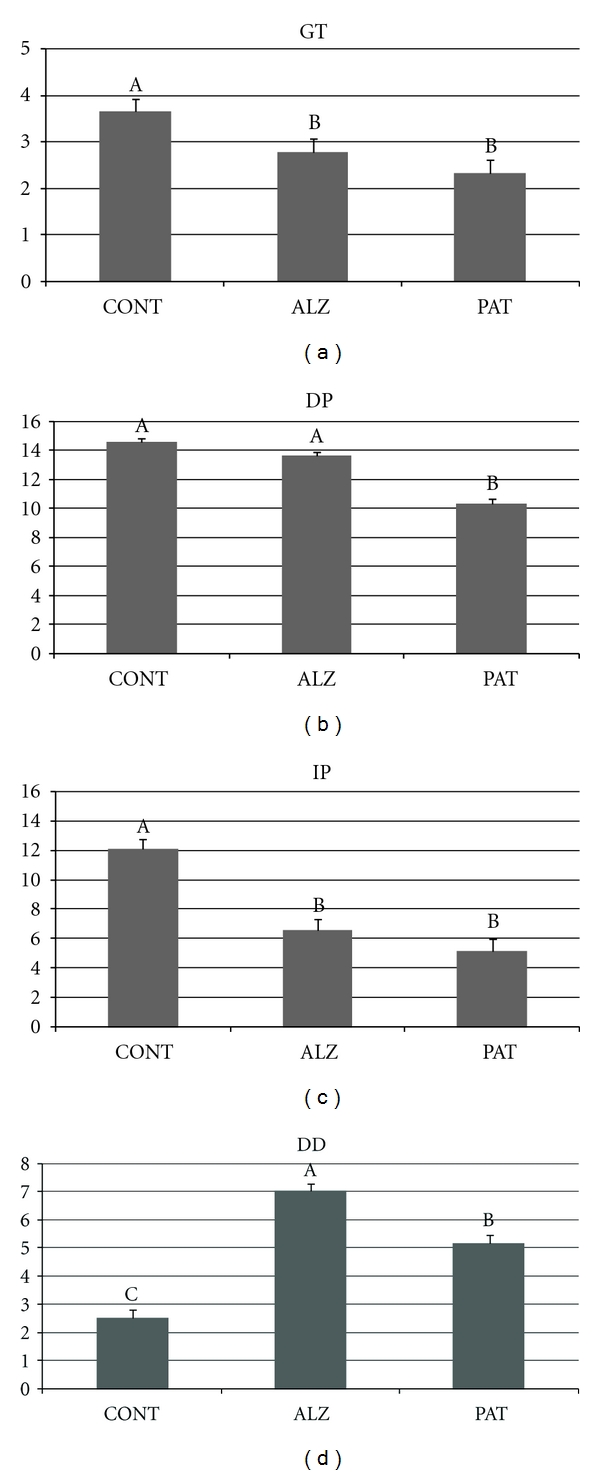
Means and standard errors of scores for gas threshold (GT), supraliminal detection (DP), identification (IP), and their difference (DD) across the 3 groups of study 2 (CONT: control elderly group, ALZ: Alzheimer's patients, PAT: smell clinic patients). Bars with the same letter do not differ significantly.

THT threshold (GT) discriminated between groups: the CONT group scored significantly higher than the PAT and ALZ groups (see Figure 2). As regards DP, it is noteworthy that ALZ patients did not score lower than the age-matched controls. Only the PAT group detected significantly fewer odorants, which is a confirmation of lower sensitivity. Identification performance (IP) discriminated elderly controls from both PAT and ALZ groups. As hypothesized, the DD difference between detection and identification scores was relevant to comparison between different causes of olfactory impairment: DD was maximum in cognitively impaired persons, the ALZ group scoring highest, and the CONT group lowest; the PAT group differed from both ALZ and CONT. The following cut-off scores may thus be suggested for DD: with a 95% confidence interval around the mean, the DD score may be [1−4[ for CONT, [4−6[ for PAT and [6−8[ for ALZ.

### 4.4. Discussion of Study 2

Screening 101 new subjects allowed various degrees of olfactory impairment to be examined. Normosmia was present in 84% of CONT group participants, 33% of the ALZ group, and 11% of the PAT group. The incidence of 16% hyposmia in the CONT group is in agreement with other studies in older adults [4].

GT, DP, IP, and DD discriminated between smell-impaired groups. GT discriminated CONT from the PAT and ALZ groups. More surprisingly, DP, which discriminated strongly between healthy and anosmic participants in study 1, only separated the PAT group from the others in study 2. One reason may be that this subtest was too easy to be able to separate different degrees of hyposmia. Identification performance (IP) uses a more difficult task to match a verbal description with perceived odor quality, resulting in larger differences between groups. The DD difference was lowest in the CONT group, although this group included 16% hyposmics. Thus, the addition of this differential score improved description of qualitative change in smell perception with normal aging and various pathologies: DD was highest in Alzheimer's patients, which can be interpreted as a feature of their dementia affecting perceptual less than cognitive processes. 

As underlined, Alzheimer patients did not score lower in detection performance than age-matched controls, but their identification scores were in the same range as for the smell-clinic patients. DD also discriminated these patients from healthy elderly controls, despite the greater age of the latter.

Comparison of the PAT group with the group of 23 “healthy” hyposmics of study 1 shows that the latter score is higher on DP and IP, which confirms that they experience a mild hyposmia. The difference between the PAT group and the anosmic group of study 1 relies mainly on DP: these patients detect more odorants than anosmic participants, which explains why their DD score is also higher.

## 5. Conclusion

Altogether, 525 subjects contributed to the validation of the LCOT. Elderly participants were well represented in this sample. The incidence of smell deficit was estimated at 5.6% in the healthy population of study 1 and at 16% in the elderly control group of study 2. Because the samples were made up of volunteers, it is difficult to generalize these figures to the French population as a whole, but they were in the same range as in other studies [37]. That 16% of subjects over 60 years of age were found to be hyposmic is, however, questionable: a number of illnesses and medications are known to impair olfaction [38, 39]. A norm for healthy aging should consider only participants in very good health and without medication; such a procedure strongly reduces the incidence of smell deficit accompanying normal aging (presbyosmia) [26]. No effect of smoking was found in the present as in other studies; sex, however, is frequently found to influence performance [5, 6]. Because sex differences mainly concern the verbal performance of women [35], the present absence of sex effect may be due to the identification task of the LCOT being rather easy, inducing a ceiling effect.

The present study sought to segregate one olfactory loss from another, whereas most olfactory tests cumulate subtask scores. In agreement with Cain et al. [40], the present results show that detection and identification, when dissociated, provide different cues for screening the severity of smell deficit. Supraliminal detection in particular emerged as a simple tool to classify subjects as anosmic, hyposmic or normosmic. The DD differential also discriminated between different smell pathologies.

## Figures and Tables

**Table 1:** List of odorants, origins, and odors. Eur: Euracli; Sig: Sigma-Aldrich.

Odorant	Origin	Odor
1,8 cineole	Sig	Eucalyptol
R-(+)-carvone	Sig	Carvi
*ω*-pentadecalactone	Sig	Musk
Tetra-hydro- thiophene	Eur	Main gas
Anise essential oil	Eur	Anise
Apple essential oil	Eur	Apple
Cinnamon essential oil	Eur	Cinnamon
Domestic fuel-oil	Total	Fuel-oil
Garlic essential oil	Eur	Garlic
Grass aroma	Eur	Grass
Lavender essential oil	Eur	Lavender
Lemon essential oil	Eur	Lemon
Mint essential oil	Eur	Mint
Orange essential oil	Eur	Orange
Smoked fish aroma	Eur	Smoked fish
Vanilla essential oil	Eur	Vanilla
Violet essential oil	Eur	Violet

**Table 2:** Distribution of the 407 healthy participants by age group.

	Age group (years)							
	<20	20–29	30–39	40–49	50–59	60–69	70–79	≥80
*N*	13	129	44	58	37	50	45	31
% Women	62%	65%	55%	45%	59%	60%	62%	81%

**Table 3:** Mean scores and standard deviations for 407 healthy participants.

Carvone threshold (CT)	3.36 ± 1.24
Gas threshold (GT)	3.97 ± 0.87
Detection performance (DP)	15.5 ± 0.9
Identification performance (IP)	14.1 ± 2.0
Difference (DD = DP − IP)	1.47 ± 1.74

**Table 4:** Influence of age on subtest scores (Fisher statistic).

Variable	B	*F*-statistic	*P*-value
CT	−0.004	2.6	0.10
GT	−0.001	0.53	0.47
DP	−0.01	24.2	<0.0001
IP	−0.05	145	<0.0001
DD	0.04	119	<0.0001

**Table 5:** Decision table for GT, DP, IP, and DD.

Anosmics	Hyposmics	Normosmics
GT < 3		
DP ≤ 7	8 to 13	for age ∈ [15 : 71], DP ≥ 14
	8 to 12	for age ∈ [72 : 91], DP ≥ 13
IP ≤ 5	6 to 12	for age ∈ [15 : 64], IP ≥ 13
	6 to 11	for age ∈ [65 : 75], IP ≥ 12
	6 to 8	for age ∈ [76 : 91], IP ≥ 9
DD ≤ 5	DD ≥ 3	for age ∈ [15 : 61], DD < 3
	DD ≥ 5	for age ∈ [61 : 71], DD < 5
	DD ≥ 8	for age ∈ [71 : 91], DD < 8

**Table 6:** Classification of the population resulting from the cutoff criteria.

	Anosmics	Hyposmics	Normosmics	Total
Normosmic sample	0	23	384	407
Anosmic sample	16	1	0	17
